# Impact of adverse childhood experiences on sensory thresholds in adults living with multimorbidity and chronic pain (the ACE-MAP study): protocol for an observational feasibility study

**DOI:** 10.1136/bmjopen-2024-091053

**Published:** 2025-01-07

**Authors:** Dhaneesha N S Senaratne, Blair H Smith, Timothy G Hales, Louise Marryat, Lesley A Colvin

**Affiliations:** 1 Chronic Pain Research Group, School of Medicine, University of Dundee, Dundee, UK; 2 Institute of Academic Anaesthesia, School of Medicine, University of Dundee, Dundee, UK; 3 School of Health Sciences, University of Dundee, Dundee, UK

**Keywords:** Chronic Pain, Multimorbidity, PAIN MANAGEMENT

## Abstract

**Abstract:**

**Introduction:**

Exposure to adverse childhood experiences (ACEs) is associated with a range of poor long-term health outcomes, including multimorbidity and chronic pain. Epidemiological evidence underpins much of this relationship; however, psychophysical testing methods, such as quantitative sensory testing (QST), may provide valuable insights into potential mechanisms. Previous studies have shown inconsistent links between ACEs and QST, but the QST profiles of people with multimorbidity have not been reported. We hypothesise that exposure to ACEs is associated with lowered QST thresholds (ie, experience of pain with milder stimuli) and that this association is stronger in adults with multimorbidity and/or chronic pain. The ACE-MAP study is a cross-sectional feasibility study with the primary aim of assessing the feasibility and acceptability of the proposed study procedures. The secondary aim is to generate preliminary data to understand the impact of ACEs on QST thresholds.

**Methods and analysis:**

We plan to recruit 40 participants, with 10 in each of the following groups: (1) chronic pain with multimorbidity; (2) chronic pain without multimorbidity; (3) multimorbidity without chronic pain; and (4) controls. Participants will complete a series of questionnaires (including on ACEs, chronic pain and long-term conditions) and will then take part in QST assessments. The primary study outcomes will include measures of feasibility and acceptability of the proposed study design. The secondary study outcomes will include exploratory analysis on the relationship between ACEs and QST thresholds.

**Ethics and dissemination:**

The study was approved by the Scotland B Research Ethics Committee (reference: 24/SS/0031). Results from the study will be presented at scientific conferences, published in a peer-reviewed journal and shared with patients and members of the public through other media streams.

**Trial registration number:**

ISRCTN10049430.

**STRENGTHS AND LIMITATIONS OF THIS STUDY**

- This study was designed with active input from our patient and public involvement group, which consists of eight individuals with lived experience of adverse childhood experiences (ACEs), multimorbidity and chronic pain.
- The questionnaire on ACEs that will be used has been co-developed by members of the Consortium Against Pain Inequality and our patient and public involvement group. It covers a broad range of ACEs and is designed to be more acceptable to participants than existing questionnaires.
- This study will use both subjective and objective assessments of pain.
- This study is limited to participants who can speak English, so that their psychological and physical safety can be ensured by the study team during the completion of questionnaires and sensory testing.
- This is an exploratory study and therefore it will have a small sample size.

## Introduction

 Adverse childhood experiences (ACEs) are potentially stressful events or environments that occur before the age of 18. They may be subdivided into forms of abuse (eg, physical), neglect (eg, emotional), household challenges (eg, parental separation) and external challenges (eg, bullying). These experiences are remarkably common: a recent meta-analysis suggested that 61% of adults have experienced at least one type of ACE, while 16% have experienced four or more.^1^ In recent years, ACEs have been the focus of many epidemiological studies that have linked them to poor outcomes in a range of physical health, mental health and social parameters in adulthood.^2^,^10^ These studies have demonstrated a dose-dependent relationship: as the burden of ACE exposure increased, so too did the burden of adverse outcomes.

Some of this evidence base has linked ACE exposure with both chronic pain^10^,^13^ and multimorbidity.^2 9^ Chronic pain, defined as pain that persists or recurs for longer than 3 months, is a common problem affecting around 34% to 44% of people in the UK, with 12% of people having ‘high impact’ chronic pain that affects their ability to cope with daily activities.^14 15^ Multimorbidity, defined as the co-occurrence of two or more long-term conditions (LTCs) in the same individual, is also a common problem with a lifetime prevalence of 33%.^16^ Chronic pain and multimorbidity are closely related: many of the risk factors for chronic pain (eg, older age, female gender, deprivation) are also risk factors for multimorbidity.^17^,^19^ Furthermore, one study reported that 69% of participants with chronic pain reported another LTC (and therefore met the definition of multimorbidity), and participants with more than four LTCs were 3.6 times more likely to have chronic pain than those with no LTCs.^20^ Thus, chronic pain is often an important component of the burden of illness in people living with multimorbidity.

There are a number of potential explanations for the relationship between ACEs, chronic pain and multimorbidity. From epidemiological studies, we know that these include social vulnerability^21^ and shared genetic vulnerability.^22^ However, other study designs can provide further insights into underlying mechanisms and may help identify potential targets for intervention. Psychophysical measurements may provide additional insights: while pain is a subjective experience, it is possible to derive objective pain measurements by using validated structured techniques.

Quantitative sensory testing (QST) is a structured method of assessing sensory pathways, deriving numerical measurements from subjective sensations. QST tests can be either static or dynamic; static QST evaluates the basal state of the nociceptive system through measurement of sensory/pain thresholds, while dynamic QST evaluates mechanisms of pain processing by using more complex stimulation of the somatosensory system.^23 24^ Studies utilising static QST assessments have reported lower pain thresholds in people with a history of physical or sexual abuse^25 26^ and in those with a history of psychological trauma.^27 28^ Some studies utilising dynamic QST assessments have reported altered pain processing in people with a history of childhood trauma^29^ or ACEs,^30^ though others have reported no relationship.^31 32^ One of the challenges is that the methods of ACE assessment and the QST protocols used vary between studies.

QST is well-established in pain research in both adult and paediatric populations, but to our knowledge, the QST profiles of individuals with multimorbidity have not been investigated. Understanding how these sensory parameters may vary based on ACE exposure may provide insights into the mechanisms that underpin the associations between ACEs, multimorbidity and chronic pain that are repeatedly seen in epidemiological studies. Our hypothesis is that exposure to ACEs is associated with lowered QST thresholds (ie, experience of pain with milder stimuli) and that this association is stronger in adults with multimorbidity and/or chronic pain. This is based on the higher prevalence of chronic pain found in people who have experienced ACEs and subsequent LTCs.^20 33 34^

The ACE-MAP study is an observational feasibility study with the primary aim of assessing the feasibility and acceptability of the proposed study procedures in adult participants, including those living with multimorbidity and/or chronic pain. The secondary aim is to generate preliminary data to understand the impact of ACEs on QST thresholds.

The ACE-MAP study is part of a larger multi-institution collaboration, the Consortium Against Pain Inequality (CAPE), that is more widely looking at the relationship between ACEs and chronic pain.^35^

## Methods and analysis

### Patient and public involvement

This study has had input from members of our patient and public involvement group (the Chronic Pain Advisory Group – CPAG), who are part of CAPE.^35^ CPAG consists of eight individuals with lived experiences of ACEs, multimorbidity and chronic pain. Thus far, the group has provided feedback on the research question, the study design (including reviewing the potential participant burden), the participant-facing study materials and the provision of appropriate support for participants. Their involvement is ongoing.

### Design and setting

This is a single-site feasibility study with a cross-sectional design. Participants will be asked to attend one assessment session at Ninewells Hospital and Medical School, University of Dundee, UK.

### Recruitment

Potential participants will be identified through multiple streams:

- The Scottish Health Research Register and Biobank (SHARE) database^36^ will be used to identify and invite potential participants that fulfil the inclusion criteria (based on SHARE data). Initial contact will be made by SHARE on behalf of the research team using established SHARE protocols. Potentially interested participants, who verbally consent to hearing more about the study, will be contacted by the research team.
- Patients who are referred to chronic pain clinics in NHS Tayside are invited to consent to receive information about future research studies. From those who have opted in, potential participants that fulfil the inclusion criteria will be contacted by a member of the clinical team. Potentially interested participants, who verbally consent to hearing more about the study, will be contacted by the research team.
- We designed advertising materials (study posters, website content) with input from our patient and public involvement group. Potential participants who see these materials or who learn of the study through word of mouth can contact the research team directly.

Potential participants identified through these streams will be contacted by the research team by phone and/or email and assessed for eligibility for participation. Inclusion/exclusion criteria for participation are:

- Inclusion criteria:
  - Age ≥18 years.
- Exclusion criteria:
  - Lacks the capacity to consent to participate in the study.
  - Unable to read or speak English to a sufficient standard to complete questionnaires or to follow instructions for sensory tests.

We will recruit participants to four groups: (1) chronic pain with multimorbidity; (2) chronic pain without multimorbidity; (3) multimorbidity without chronic pain and (4) a control group (people with neither multimorbidity nor chronic pain). We will use the list of LTCs reported by Ho *et al*^37^ in a Delphi consensus study^37^ to determine multimorbidity status (table 1). The inclusion and exclusion criteria for each group are listed in table 2. During the recruitment period, if it becomes evident that there is over-representation or under-representation from any of these groups the research team will prioritise the recruitment of individuals from specific groups to ensure an appropriate mix.

**Table 1:** Long-term conditions included in the measure of multimorbidity

Body system (based on ICD-10 chapters)	Long-term condition
Cardiovascular disease	Stroke
	Coronary artery disease
	Heart failure
	Peripheral artery disease
	Heart valve disorders
	Arrhythmia
	Venous thromboembolic disease
	Aneurysm
	Hypertension (treated and untreated)
Metabolic and endocrine diseases	Diabetes
	Addison’s disease
	Cystic fibrosis
	Thyroid disorders
Respiratory disease	Chronic obstructive pulmonary disease
	Asthma
	Bronchiectasis
Neurological disease	Parkinson’s disease
	Epilepsy
	Multiple sclerosis
	Paralysis
	Transient ischaemic attack
	Peripheral neuropathy
Cancer	Solid organ cancers
	Haematological cancers
	Metastatic cancers
	Melanoma
	Benign cerebral tumours that cause disability
Mental and behavioural disorder	Dementia
	Schizophrenia
	Depression
	Anxiety
	Bipolar disorder
	Drug or alcohol misuse
	Eating disorder
	Autism
	Post-traumatic stress disorder
Musculoskeletal disease	Connective tissue disease
	Osteoarthritis
	Long-term musculoskeletal problems due to injury
	Osteoporosis
	Gout
Digestive disease	Chronic liver disease
	Inflammatory bowel disease
	Chronic pancreatic disease
	Peptic ulcer
Urogenital disorder	Chronic kidney disease
	End-stage kidney disease
	Endometriosis
	Chronic urinary tract infection
Haematological disorder	Anaemia (including pernicious anaemia and sickle cell anaemia)
Eye disease	Vision impairment that cannot be corrected
Ear disease	Hearing impairment that cannot be corrected
	Meniere’s disease
Infectious disease	HIV infection and AIDS
	Chronic Lyme disease
	Tuberculosis
	Post-acute COVID-19
Congenital disease	Congenital disease or chromosomal abnormalities

Adapted from Ho *et al*.^37^Ho et al. (2022).

ICD-10International Classification of Diseases (10th Revision)

**Table 2:** Inclusion and exclusion criteria

Chronic pain with multimorbidity group	Chronic pain without multimorbidity group	Multimorbidity without chronic pain group	Control group
Inclusion criteria: Age ≥18 years Has ≥1 of the LTCs listed in table 1 Has a diagnosis of chronic pain AND/OR has had persistent or recurring pain lasting >3 months	Inclusion criteria: Age ≥18 years Has a diagnosis of chronic pain AND/OR has had persistent or recurring pain lasting >3 months	Inclusion criteria: Age ≥18 years Has ≥2 of the LTCs listed in table 1	Inclusion criteria: Age ≥18 years
Exclusion criteria: Lacks the capacity to consent to participate in the study Unable to read or speak English to a sufficient standard to complete questionnaires or to follow instructions for sensory tests	Exclusion criteria: Lacks the capacity to consent to participate in the study Unable to read or speak English to a sufficient standard to complete questionnaires or to follow instructions for sensory tests. Has ≥1 of the LTCs listed in table 1	Exclusion criteria: Lacks capacity to consent to participate in the study Unable to read or speak English to a sufficient standard to complete questionnaires or to follow instructions for sensory tests. Has a diagnosis of chronic pain, AND/OR has had persistent or recurring pain lasting >3 months	Exclusion criteria: Lacks the capacity to consent to participate in the study Unable to read or speak English to a sufficient standard to complete questionnaires or to follow instructions for sensory tests. Has ≥2 of the LTCs listed in table 1 Has a diagnosis of chronic pain, AND/OR has had persistent or recurring pain lasting >3 months

LTC, long term condition

Recruitment will open in July 2024 and is predicted to close by the end of 2025.

### Procedure

Potential participants will be provided with a participant information sheet by email or post (depending on individual preference) prior to attending for their assessment session. They will be given at least 24 hours to consider their participation and will have the opportunity to ask questions (via email or telephone) prior to the session.

Written informed consent will be obtained at the start of the assessment session, prior to the completion of any study assessments.

Next, participants will complete a series of questionnaires on:

- Demographics, including year of birth, sex assigned at birth, ethnicity, recruitment stream, Scottish Index of Multiple Deprivation, level of education, employment status and household income.
- Health behaviours, using the General Practice Physical Activity Questionnaire^38^ and the WHO’s Alcohol, Smoking, and Substance Involvement Screening Test.^39^
- Long-term conditions, using the LTCs listed in table 1.
- Chronic pain, using the UK Biobank Pain Web Questionnaire.^40^
- Adverse childhood experiences, using the CAPE ACE questionnaire (in the final stages of development).
- Current medication.

Then participants will have their height and weight recorded.

Next, we will begin QST based on a protocol devised by the German Research Network on Neuropathic Pain,^41^ with the addition of a conditioned pain modulation (CPM) paradigm. Each test will be performed bilaterally, with the exception of the CPM test, which will be performed on one side only.

- Thermal detection and pain thresholds will be measured at the forearm or hand using a Modular Sensory Analyser with a standard 25×50 mm thermode (Somedic SenseLab, Sweden). Starting from a baseline temperature of 32°C, we will use the ramp method with a rate of change of 1°C/s. The lower and upper cutoffs will be 10°C and 50°C, respectively. Participants will be instructed to press a button at the appropriate point for each test; for the cold detection threshold and warm detection threshold, this will be when they ‘first feel a [cold/warm] sensation’ at the test site; and for the cold pain threshold and the heat pain threshold, this will be when they ‘first feel something that is painfully [cold/warm]’ at the test site. For each parameter, we will take three measurements and calculate the mean. The result of the TSL test, in which alternating cold and warm stimuli are applied, will be the number of paradoxical heat sensations reported (ie, where cold is reported as warm and vice versa).
- The mechanical detection threshold will be measured at the forearm or hand using a standardised calibrated set of von Frey filaments (Somedic SenseLab, Sweden) that exert forces of 0.63 mN to 235.36 mN on bending. With their eyes closed, participants will be asked to verbally report when they feel something touch the test site. Using the method of limits, we will take five detection threshold measurements and report the geometric mean.
- The mechanical pain threshold will be measured at the forearm or hand using calibrated weighted PinPrick stimulators (MRC Systems, Germany) that exert forces from 8 mN to 512 mN. With their eyes closed, participants will be asked to verbally report when they feel something sharp at the test site. Using the method of limits, we will take five pain threshold measurements and report the geometric mean.
- Stimulus response functions will be measured at the forearm or hand using the same PinPrick stimulators as described above and three light tactile stimulators: cotton wisp exerting a force of ~3 mN, a cotton wool tip attached to an elastic strip exerting a force of ~100 mN and a standardised brush (Somedic SenseLab, Sweden) exerting a force of ~300 mN. Stimuli will be applied in a pseudorandom sequence (consistent across all participants) with approximately a 10 s interval between stimuli. With their eyes closed, participants will be asked to verbally report a pain score for each stimulus on a numerical rating scale of 0 to 100. Mechanical pain sensitivity will be calculated as the geometric mean of the pain scores for all pinprick stimuli. Dynamic mechanical allodynia will be calculated as the geometric mean of the pain scores for all light tactile stimulators.
- Wind-up ratio (WUR) will be measured at the forearm or hand using the 256mN PinPrick stimulator. A single stimulus will be applied followed by a series of 10 stimuli to the same area. With their eyes closed, participants will be asked to verbally report two pain scores on a numerical rating scale of 0 to 100, one for the single stimulus and one for the series of 10. We will repeat this five times. The WUR will be calculated as the mean pain score for the series divided by the mean pain score for the single stimulus.
- The vibration detection threshold (VDT) will be measured at a bony prominence at the wrist or elbow using a 64 Hz Rydel-Seiffer graded tuning fork (Ragg Tuning Forks, UK). With their eyes closed, participants will be asked to verbally report when they feel the vibration sensation stop at the test site. We will take three measurements and calculate the mean.
- The pressure pain threshold (PPT) will be measured at the thenar eminence with an algometer (Somedic SenseLab, Sweden). Participants will be asked to verbally report when they ‘first feel pain’ at the test site. We will take three measurements and calculate the mean.
- CPM will be measured using the cold pressor test. The participant will be asked to lower their non-dominant hand into a cold water bath at 10°C±2°C (the conditioning stimulus). The water temperature will be generated by a Grant LTC4 refrigerated circulating bath (Grant Instruments, UK). After 20 s, we will measure two modalities of test stimuli on the dominant arm; PPT followed by HPT, as described above. We will take three measurements of each and calculate the mean. CPM will be reported as an absolute value (the mean PPT or HPT prior to the conditioning stimulus minus the mean PPT or HPT during the conditioning stimulus) and as a percentage change (absolute CPM divided by the mean PPT or HPT prior to the conditioning stimulus). Thus, a negative CPM value will indicate CPM-induced pain inhibition.^42^ We will also measure the duration of time that the hand was submerged in the cold water bath (up to a maximum of 120 s) and ask the participant to verbally report a pain score for the water bath on a numerical rating scale of 0 to 100.

Finally, participants will complete a questionnaire on study acceptability, using a questionnaire adapted from Sekhon *et al*.^43^

There is no planned follow-up for this study.

### Sample size calculation

This is a feasibility study, and therefore a formal sample size calculation is not required. However, preliminary data from this stage will be used to determine the sample size required for a future definitive study. Whitehead *et al* (2016) suggested that a sample size of 10 per treatment arm in the pilot phase would provide enough preliminary data to determine the sample size needed to detect a medium effect in a full interventional trial.^44^ While this study is not interventional, we believe the principles to be transferable, and therefore, we will aim to recruit 40 study participants, with 10 in each group.

### Statistical analysis

The primary aim of the ACE-MAP study is to assess the feasibility and acceptability of the proposed study procedures. Recruitment feasibility will be assessed per stream by calculating the overall recruitment rate (number of participants successfully recruited to the study per week) and the recruitment proportion (number of participants successfully recruited to the study divided by the number of potential participants approached). Acceptability will be assessed with a modified version of an acceptability questionnaire developed by Sekhon *et al*^43^ administered to study participants at the end of the assessment session. The data will be presented as counts and proportions, with free-text comments presented as either themes or original anonymised quotes.

The secondary aim is to generate preliminary data to understand the impact of ACEs on QST thresholds in people with multimorbidity and/or chronic pain. Descriptive statistics will be generated on all groups of data (demographics, LTCs, chronic pain, ACEs, medication and QST parameters). We will perform exploratory analyses, such as investigating the association between the number of ACEs experienced and individual QST parameters, and how these relationships vary based on multimorbidity/chronic pain status, using a range of statistical techniques (eg, χ^2^ tests, Student’s t-tests, regression analysis). These analyses will inform the project design and sample size calculation for a subsequent definitive study.

All statistical analyses will be performed in R V.4.2.2 using the RStudio integrated development environment (RStudio Team, Boston, USA).

### Ethics and dissemination

Ethical approval for the ACE-MAP study was granted by the National Health Service Scotland B Research Ethics Committee (reference: 24/SS/0031). ACEs are a sensitive and potentially triggering subject, and so care will be taken to ensure participant safety. The ACE questionnaire that we will use is based on the WHO’s Adverse Childhood Experiences International Questionnaire^45^ and has been adapted by CAPE with detailed input from our patient and public involvement group. This included ensuring that the questionnaire wording is respectful, acceptable to people who have experienced ACEs (and those who have not) and that a broad and relevant range of ACEs are covered to reflect a range of backgrounds. Our patient and public involvement group highlighted the need to signpost to organisations that can provide additional support; these details will be offered to each participant.

The ACE-MAP study has been registered in the ISRCTN registry (ID: ISRCTN10049430). It will be conducted in compliance with the principles of the Declaration of Helsinki (2013) and Good Clinical Practice.

Results from the study will be presented at scientific conferences, published in a peer-reviewed journal and shared with patients and members of the public through other media streams.
