# Supplementary material for: Impact of adverse childhood experiences on sensory thresholds in adults living with multimorbidity and chronic pain (the ACE-MAP study): protocol for an observational feasibility study
Source: BMJ Open. 2025 Jan 7;15(1):e091053. doi: 10.1136/bmjopen-2024-091053 (PMC11749611; doi:10.1136/bmjopen-2024-091053)
Supplement: Supplementary file 1 [file bmjopen-2024-091053.reviewer_comments.pdf]

PEER REVIEW HISTORY

BMJ Open publishes all reviews undertaken for accepted manuscripts. Reviewers are asked to complete a checklist review form and are provided with free text boxes to elaborate on their assessment. These free text comments are reproduced below.

ARTICLE DETAILS

Title (Provisional)

The impact of Adverse Childhood Experiences on sensory thresholds in adults living with Multimorbidity And chronic Pain (the ACE-MAP study): protocol for an observational feasibility study

Authors

Senaratne, Dhaneesha Navin Sannasgala; Smith, Blair Hamilton; Hales , TG; Marryat, Louise; Colvin, Lesley

---

VERSION 1 - REVIEW

**Reviewer**	**1**
**Name**	**Vasquez, Namrata**
**Affiliation**	**Houston Methodist Hospital, Psychiatry & Behavioral Health**
**Date**	**23-Sep-2024**
**COI**	**None**

Authors propose a protocol examining potential pain processes that underly and/or contribute to multimorbidity with and without chronic pain, including implementation of QST (including CPM). Examination of the role of ACES in multimorbidity is important, particularly in terms of advancing knowledge of how trauma may impact our psychophysiological development, risk factors, and disease. Some recommendations for advanced clarity and background are presented:

- While the multimorbidity with/without chronic pain sample is important, and novel, researchers have explored the relationship between ACEs and QST, including CPM paradigms. Mention of the existing work and consequent findings in the Abstract is imperative to present the background of this work, and what it would add to the literature.

- CPM is discussed as separate from QST, but is in fact one type of QST modality (https://www.sciencedirect.com/science/article/abs/pii/S0149763424000812). CPM should be discussed as one type of quantitative sensory testing, rather than a distinct form of testing. Rather, CPM is a phenomenon or process that occurs and can be measured using QST

(https://pubmed.ncbi.nlm.nih.gov/33334645/#:~:text=Conditioned%20pain%20modulation%20(CPM)%20is,well%20as%20the%20inhibitory%20effect.)

- It would be helpful to discuss existing frameworks that discuss multimorbidity, such as the shared vulnerability and/or mutual maintenance model that have been developed to explain the relationship between PTSD and pain, for example.

- The statistics section could be strengthened by listing specific analyses that will be implemented, and method of doing so (e.g., R, statistical software), and if statistical consultation will take place. For example, "exploratory analyses" is not sufficiently specific.

- In the Methods section, at multiple points, "take measurements of threshold" is described but it is unclear, and therefore not replicable, how these measurements will be taken. Will patients verbally let you know, or press a button when they first feel pain? How are they instructed to respond to the stimuli? Please add detail.

- Please include when eligibility will be assessed in the procedures section.

**Reviewer**	**2**
**Name**	**Harris, Jane**
**Affiliation**	**Liverpool John Moores University, Public Health Institute**
**Date**	**04-Nov-2024**
**COI**	**None**

Thank you for the opportunity to review this study protocol for a feasibility study exploring the impact of ACEs on sensory thresholds among adults with mulitmorbidity and chronic pain. I have only minor comments

- is there an age inclusion/exclusion criteria for the study

- if possible it would be good to have some Scotland specific data in the introduction to justify the study setting

- are there ay exclusion criteria for participation in CPM or QST testing

---

VERSION 1 - AUTHOR RESPONSE

Reviewer #1:

"While the multimorbidity with/without chronic pain sample is important, and novel, researchers have explored the relationship between ACEs and QST, including CPM paradigms. Mention of the existing work and consequent findings in the Abstract is imperative to present the background of this work, and what it would add to the literature."

We agree and so have added a series of references (Introduction -> paragraph 4) to highlight this important background work (Nanavaty et al. 2023 [28], Sherman et al. 2015 [29], Thomas et al. 2024 [30], Rassu et al. 2020 [31], Morris et al. 2023 [32]).

We have also amended the abstract accordingly.

"CPM is discussed as separate from QST, but is in fact one type of QST modality"

Thank you for highlighting this point. We have rewritten all relevant sections of the manuscript to reflect this.

We have also added detail on static vs dynamic QST (Introduction -> paragraph 4) to help present the various forms of QST in a structured way, and have added two new references to support this (Marcuzzi et al. 2017 [23], van Driel et al. 2024 [24]).

"It would be helpful to discuss existing frameworks that discuss multimorbidity, such as the shared vulnerability and/or mutual maintenance model that have been developed to explain the relationship between PTSD and pain, for example."

Thank you for this suggestion. We agree that there are likely to be a number of interesting models to explain how multimorbidity, chronic pain, and specific conditions like PTSD are linked. We have added additional detail to highlight this (Introduction -> paragraph 4) and have also included two new references (Nguyen et al. 2019 [21], Westergaard et al. 2024 [22]). However, this is a complex area and we feel that entering into a detailed discussion on this subject is beyond the scope of this protocol manuscript for a feasibility study.

"The statistics section could be strengthened by listing specific analyses that will be implemented, and method of doing so (e.g., R, statistical software), and if statistical consultation will take place. For example, "exploratory analyses" is not sufficiently specific."

We have added information on the statistical tests and software to be used (Methods and analysis -> Statistical analysis -> paragraphs 2 and 3).

This is a protocol manuscript for a feasibility study, and so the primary aim is to assess our study procedures. Any analysis on the data would be hypothesis generating, not hypothesis testing. This is why we chose to use the phrase "exploratory analyses". However, we have expanded on this phrase to explain in more detail on the purpose of this analysis – i.e. to inform the project design and sample size calculation for a subsequent definitive study. (Methods and analysis -> Statistical analysis -> paragraph 2).

"In the Methods section, at multiple points, "take measurements of threshold" is described but it is unclear, and therefore not replicable, how these measurements will be taken. Will patients verbally let you know, or press a button when they first feel pain? How are they instructed to respond to the stimuli? Please add detail."

We have added this information for each test (Methods and analysis -> Procedure -> paragraph 5).

"Please include when eligibility will be assessed in the procedures section."

We have added this information (Methods and analysis -> Recruitment -> paragraph 2).

Reviewer #2:

"is there an age inclusion/exclusion criteria for the study"

This information was listed in Table 2, however we now realise that it was not clear. We have therefore added this to the main text as well (Methods and analysis -> Recruitment -> paragraph 2).

"if possible it would be good to have some Scotland specific data in the introduction to justify the study setting"

Thank you for this suggestion. We agree that it is helpful to place the study within the geographical context and for this reason, we have reported a range of UK prevalence figures for chronic pain and multimorbidity. Reference 14 (Fayaz et al. 2016) is a systematic review and meta-analysis on the prevalence of chronic pain and one of its important contributing papers (Elliot et al. 1999) describes the prevalence of chronic pain across a large cohort in the Grampian region of Scotland. Similarly, reference 16 (Nguyen et al. 2019) is a systematic review and meta-analysis of the prevalence of multimorbidity and one of its contributing papers (Johnston et al. 2019) describes the prevalence of multimorbidity in the Aberdeen Children of the 1950s cohort. Hopefully the inclusion of both of these papers in their respective reviews will ensure that the introduction is representative of the Scottish population, whilst also being more generalisable to the rest of the UK.

"are there ay exclusion criteria for participation in CPM or QST testing"

As for point 7 above, this information was listed in Table 2, however we now realise that it was not clear. We have therefore added this to the main text as well (Methods and analysis -> Recruitment -> paragraph 2).

We look forward to your evaluation of our revised manuscript.

VERSION 2 - REVIEW

**Reviewer**	**1**
**Name**	**Vasquez, Namrata**

Affiliation **Houston Methodist Hospital, Psychiatry & Behavioral Health**

Date **10-Dec-2024**

COI

I appreciate the authors' thoughtful response and incorporation of relevant feedback as they saw needed. One comment I have is that recruitment reportedly began in July 2024, it may be helpful to note updates since July if relevant as per BMJ Open's guidelines, if editor believes this is important.
